# Enhancement growth, water use efficiency and economic benefit for maize by drip irrigation in Northwest China

**DOI:** 10.1038/s41598-023-35611-9

**Published:** 2023-05-24

**Authors:** Mengjie Liu, Fei Liang, Quansheng Li, Guodong Wang, Yuxin Tian, Hongtao Jia

**Affiliations:** 1 College of Resources and Environment, Yi Li Normal University, Yi Li, Xinjiang, 835000 China; 2 College of Resources and Environment, Xinjiang Agricultural University, Urumqi, 830052 China; 3 Institute of Farmland Water Conservancy and Soil-Fertilizer, Xinjiang Academy of Agricultural Reclamation Science, Shihezi, 832000 China; 4 School of Water Conservancy and Construction Engineering, Shihezi University, Shihezi, 832000 China; 5 College of Grassland, Xinjiang Agricultural University, Urumqi, 830052 China

**Keywords:** Environmental sciences, Hydrology

## Abstract

The application of drip irrigation has been paid more and more attention, but there was lack of systematic comparative analysis between drip irrigation and conventional border irrigation method for maize, currently. A 7-year field study from 2015 to 2021 evaluated the effects of drip irrigation (DI, 540 mm) or conventional border irrigation method (BI, 720 mm) on maize growth, water use efficiency (WUE) as well as profitability. The results showed the plant height, leaf area index, yield, WUE and economic benefit of maize with DI had significantly higher than BI. The dry matter translocation, the dry matter transfer efficiency and contribution of dry matter translocation to grain with DI showed significant increase of 27.44%, 13.97% and 7.85% compared to BI, respectively. In comparison to conventional border irrigation, the yield of drip irrigation increased by 14.39%, as well as WUE and irrigation water use efficiency (IWUE) increased by 53.77% and 57.89%. The net return and economic benefit of drip irrigation was 1998.87 and 756.58 USD$ hm^−1^ higher than that of BI. Drip irrigation increased net return and benefit/cost ratio by 60.90% and 22.88% compared with BI. These results demonstrate that the drip irrigation can effectively improve the growth, yield, WUE and economic benefit of maize in northwest China. Therefore, drip irrigation can be used for maize cultivation to increase crop yield and WUE in northwest China, which has cut down on irrigation water about 180 mm.

## Introduction

The distribution of water resources in China is uneven, which is manifested in the rich southeast and the lack of northwest. The shortage of water resources has become an important factor restricting the development of agriculture in northern China^1^. At present, China's agricultural water consumption accounts for more than 70% of total water consumption, and irrigation water accounts for 90–95% of agricultural water consumption^2^. For the northwest region with relatively less water resources, cotton, wheat and maize are the main crops. The crops growth is closely related to water availability^3^. How to make economic and effective use of water resources and implement reasonable irrigation measures are the core issues of agricultural production^4,5^. Therefore, it is imperative to optimize irrigation method to improve WUE.

Due to difference of various irrigation technologies, a significant part of agricultural research is focused on improving WUE and conserving water without yield penalties^6^. Conventional border irrigation has high irrigation quota, poor uniformity, difficult to control, and large field evaporation, which makes it difficult to further improve yield and WUE^7^. Drip irrigation technology is a new type of surface irrigation technology for the development of water-saving agriculture^8^, which has proven to be successful in saving water and improving yields. Suryavanshi et al.^9^ found that drip irrigation increased wheat yields compared to sprinkler and pool irrigation. Zhang et al.^10^ and Liu et al.^11^ found that compared with furrow irrigation, drip irrigation at-30 kPa enhanced yield by 4.3–15%, increased net profit by 3.1–23%, and reduced water application by 57%. Raina et al.^12^ found that drip irrigation besides giving a saving of 54% irrigation water resulted in 40% higher fruit yield compared with the surface irrigation. Due to the different irrigation amount and irrigation intensity of different water-saving irrigation techniques^13^, irrigation method directly affects the growth and development of maize^14^. Qin et al.^15^ research results show that under different irrigation methods, drip irrigation not only achieve the role of water saving, but also on the growth of maize can play a role in increasing production. O’Neill et al.^16^ showed that by using furrow irrigation to produce the same number of maize grains, subsurface drip irrigation saves nearly 30% of the total water consumption (irrigation, rainwater, soil water), while sprinkler irrigation saves nearly 8% of the water consumption. Mehriya et al.^17^ reported that maximum water-use efficiency 5.7 kg hm^−1^ and water saving 39.04% was observed under drip irrigation.

Until now, Irrigation research not only focuses on promoting maize growth, but also on yield, water distribution, water use efficiency, and so on. There are few long-term comparative studies on drip irrigation and conventional border irrigation in Northwest China. The lack of long-term research cannot systematically explain how much benefit has been brought to maize production in northwest China since water-saving irrigation. We assume that drip irrigation has the advantages of promoting maize growth, improving WUE and economic benefits, compared with conventional border irrigation. Much of these previous works were quantitative assessments conducted of short-term effects (≤ 2 years) and were limited to a specific aspect (soil conditions or crop characteristics). For Xinjiang, China, there are no published results of long-term drip irrigation versus conventional border irrigation for maize. In this paper, aiming at the same research area, the effects of drip irrigation and conventional border irrigation on maize growth, IWUE and economic benefit were studied by 7 years of comparative experiments under the same soil texture and planting mode. Therefore, this analysis provides a scientific basis for the promotion and application of water-saving irrigation methods for maize planting and a theoretical basis for the sustainable development of agriculture in arid areas.

## Material and methods

### Experiment site

**Figure 1:**
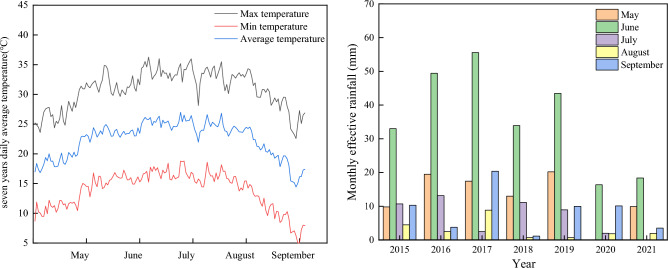
Meteorological variation during maize growth periods from 2015 to 2021. (**a**) Daily average temperature. (**b**) Monthly effective rainfall.

Field experiments were conducted in the Crop Water Use Experiment Station of the Ministry of Agriculture in Shihezi City, northern China (86° 09′ E, 45° 38′ N) from 2015 to 2021. The region has a temperate continental climate, with an annual average sunshine time of about 2770 h. The accumulated temperature above 10 °C is 3649 °C. The average annual rainfall is 125.0–207.7 mm, and the average annual evaporation is 1942 mm. The maximum/minimum temperatures and mean precipitation for the growth season in 7 years during the maize growth periods are shown in Fig. 1. The groundwater depth varies from 2 to 3 m in different years. The soil type is gray desert soil. Seven-year averages of the soil’s physicochemical properties are shown in Table 1.

**Table 1:** The physicochemical properties of soil in the test site.

Soil depth (cm)	Organic matter (g kg^−1^)	Total nitrogen (g kg^−1^)	Olsen-P (mg kg^−1^)	Avail. K (mg kg^−1^)	Bulk density (g cm^−3^)	Saturated volumetric water content (%)	pH
0–20	16.79	1.44	26.52	415.98	1.56	32.01	7.71
20–40	17.92	1.40	26.76	416.78	1.67	33.14	7.83
40–60	16.74	1.38	23.56	354.65	1.72	33.26	7.96
60–80	8.16	1.03	8.13	246.37	1.74	34.54	8.14
80–100	7.04	0.80	6.15	214.47	1.76	35.67	8.16

### Experimental design

A field experimental design consisting of three replicates were used in the study, with drip irrigation (DI) and conventional border irrigation (BI). The irrigation quota of drip irrigation was 540 mm, while the irrigation quota of conventional border irrigation was 720 mm, which refer to the local farmer irrigation quantity. Drip irrigation uses an integrated water and fertilizer irrigation model. The conventional border irrigation, the seedling water is sown with seed fertilizer, and the later irrigation adopts the simple water and fertilizer integration mode. The irrigation and fertilization levels in each growth period and the whole growth period is shown in Table 2. Drip irrigation maize sowing, harvesting, and sampling time are shown in Table 3. Considering the marginal effect of different irrigation methods, the 6 plots were separated from adjacent plots by 2.2 m-wide isolation strips, and the size of each plot (110 m^2^) was 20 m long and 5.5 m wide. In each plot, water reading meter and fertilizer tank were installed to monitor the amount of irrigation water and fertilizer that were applied, respectively.

**Table 2:** Irrigation and fertilization in different periods from 2015 to 2021.

Treatment	Irrigation and fertilization/period	Seedling stage	Jointing stage	Small bell-mouth stage	Big bell-mouth stage	Heading stage	Flowering stage	Silking stage	Grain formation stage	Milk-ripe stage	Total
DI	Irrigation quantity (mm)	16.4	69.1	69.1	69.1	69.1	69.1	69.1	65.5	43.5	540.0
	Urea (kg hm^−2^)	0.0	81.8	81.8	90.9	81.8	81.8	72.7	54.5	0.0	545.3
	Monoammonium phosphate (kg hm^−2^)	36.4	36.4	45.5	45.5	45.5	27.3	18.2	18.2	0.0	273.0
	Potassium sulphate (kg hm^−2^)	0.0	18.2	27.3	27.3	36.4	22.7	18.2	13.6	0.0	163.7
BI	Irrigation quantity (mm)	–	184.8		184.8		184.8		165.6		720.0
	Urea (kg hm^−2^)	–	163.6		172.7		154.5		54.5		545.3
	Monoammonium phosphate (kg hm^−2^)	–	81.8		90.9		45.5		18.4		273.0
	Potassium sulphate (kg hm^−2^)	–	45.5		63.6		40.9		13.7		163.7

**Table 3:** Maize sowing, harvesting, and sampling time.

Years	Sowing date	Harvest date	Flowering stage	Maturity stage
2015	2nd May	25th September	15th July	24th August
2016	30th April	24th September	17th July	25th August
2017	7th May	28th September	20th July	28th August
2018	28th April	27th September	18th July	25th August
2019	30th April	22nd September	14th July	22nd August
2020	26th April	1st October	15th July	2nd September
2021	7th May	24th September	19th July	27th August

**Figure 2:**
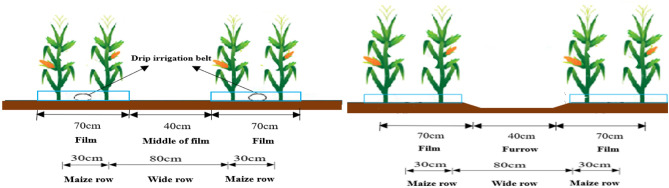
Diagram of drip irrigation and conventional border irrigation for maize cultivation.

A joint planter was used to lay drip tapes, plastic film and sow. Its planting density was 1.26 × 10^5^ hm^−2^ in the experimental. The plants were sown with alternating wide and narrow rows of 0.8 m and 0.3 m, and the spacing between plants within a row was 14.4 cm, respectively (Fig. 2, the spacing between the drip tapes was 110 cm). Pest and weed control followed the conventional practices in the area.

### Material

The maize variety “ZD958”, which is commonly planted in northern China, was used as the experimental variety. Zhengdan 958 was the offspring of inbred Zheng 58 and Chang 7-2 (deposition number 20000009), which are approved in China. In this study, the seeds of Zhengdan 958 were provided by Beijing Denong Seed Technology Co. Ltd. Experimental research and field studies on plants complied with relevant institutional, national, and international guidelines and legislation. Urea (N ≥ 46.4%, granules) used in the experiment was produced by Xinlianxin Co, Ltd. (Xinjiang, China). Monoammonium phosphate (N ≥ 12%, P_2_O_5_ ≥ 61%, powder) is produced by Guizhou Kai Phosphorus Group Co., Ltd. (Guiyang, China). Potassium sulfate is produced by Luobupo Potassium Salt Co., Ltd. (Xinjiang, China).

The source of irrigation water was a deep well with a depth of 100 m; the salinity of the water was 0.2–0.3 g L^−1^. The type of drip irrigation belt was a single wing labyrinth drip-irrigation belt (WDF16/2.6–100) produced by Xinjiang Tianye Company (Shihezi, China). The wall thickness was 0.18 mm, the inner diameter was 16 mm, the drip hole spacing was 300 mm, the rated flow was 2.0 L h^−1^, and the working pressure was 0.1–0.15 MPa.

## Sampling and measurements

### Stand growth index

#### Plant height

Ten maize plants with the similar growth were randomly selected from each treatment at flowering and maturity stages, and the height from the ground to the top of the maize plants was measured by tape^18^.

#### Leaf area index (LAI)

Ten representative plants were selected from the central rows of each plot to determine the green leaf area (GLA) non-destructively at flowering and maturity stages. Leaf length (L) and maximum width (W) were recorded and used to calculate GLA.

$$GLA = 0.75\times L\times W$$

$$LAI = GLA\times N/S$$

where N is the number of plants within a unit area of land and S is the unit area of land^19^.

#### Soil and plant analyzer development (SPAD)

The SPAD-502 chlorophyll meter (*Minolta, JPN*) was used to determine the ear leaves of ten maize plants randomly and continuously selected at flowering and maturity stages.

#### Dry matter determination

At flowering and maturity of maize, four maize plants of uniform growth were randomly selected from each treatment, cut from the bottom of the stems of the plants with scissors, and the leaves, stems, and reproductive organs^20^ at a later stage were put into archival bags and weighed in fresh weight, after which the weight of dry matter was measured using the drying and weighing method. All the plant samples were heated for 30 min at 105 °C and then dried at 85 °C to a constant weight. Each plant fraction was weighed to obtain its dry-matter weight.

Field sampling and investigation were conducted at the flowering stage (15th July, 2015, 17th July, 2016, 20th July, 2017, 18th July, 2018, 14th July, 2019, 15th July, 2020, and19th July, 2021) and maturity stage (24th August, 2015, 25th August, 2016, 28th August, 2017, 25th August, 2018, 22nd August, 2019, 2nd September, 2020, and 27th August, 2021) of maize.

### Yield and its components

During the maize maturity period, random sampling was done for each plot. Twenty maize plants were chosen continuously at each point, and the length of the panicles, the number of rows, and the length of the baldness was measured, and then the ears of the maize were threshed. The grain was air-dried and weighed (called its 1000-grain mass and total grain mass), then converted into yield per hectare. Grain yield and kernel weight were expressed at 14% moisture content.

### Data analysis

Yield (kg hm^−2^) = 20 grain weight (g)/20 panicles × 126,000/1000 × [1 − grain moisture content (%)]/(1 − 14%)^21^.

Dry matter translocation (kg hm^−2^) = stem and leaf dry matter at flowering stage − stem and leaf dry matter at maturity stage;

Dry matter transfer efficiency (%) = dry matter translocation/stem and leaf dry matter at flowering stage ×100;

Contribution of dry matter translocation to grain (%) = dry matter translocation/grain yield × 100^22^.

### Water use efficiency (WUE)^23^

Seasonal evaporate–transpiration (ET) was estimated using water balance approach

$$ET = \, P + \, I \, + \, C_{p} - \, D_{p} - R_{f} -\Delta S$$

where P, precipitation; I, irrigation; C_p_, contribution through capillary rise from groundwater; D_p_, deep percolation; R_f_, runoff; ΔS = S_f_ − S_i_, change in the soil water storage in the profile; where S_i_, soil water storage in the profile at sowing and S_f_, soil water storage in the profile at harvest.

Due to the depth of groundwater between 2 and 3 m, C_p_ was assumed negligible. D_p_ was considered negligible beyond 90 cm because of negligible changes in the soil moisture storage below 90 cm soil depth. There was no run off (R_f_) from the field as all the plots were provided with bunds. ΔS, the soil water storage at sowing time is similar to that at harvest time, which can be ignored. Thus,

$$ET = P + I$$

$$WUE = Y/ET$$

where Y is the grain yield of maize.

Calculation formula of irrigation water use efficiency^24^ (kg m^−3^) is

$$IWUE = Y/I$$

### Economic benefits analysis

In order to simply compare the economic benefits of drip irrigation and conventional border irrigation, the annual land rent, machinery, seed, pesticides, insurance, labor, and fertilizer were set at the same price. The net income per hectare of all treatments were calculated by subtracting the planting costs from the total income. The ratio of benefits to costs (B:C) was calculated using the formula in equation^25^:

Benefit/cost ratio = gross returns (USD$ hm^−1^)/cost of cultivation USD$ hm^−1^

### Statistical analysis

The paper uses 7 years average data. All data were statistically analyzed using SPSS 25.0, including one-way ANOVA, multiple mean comparison using the least significant difference (LSD) test (α = 0.05). The figures were prepared via origin 2018 and excel 2016. Duncan’s test was performed to conduct multiple comparisons to identify significant differences between the means of different treatments. Differences were considered statistically significant when p < 0.05.

## Results

### Growth parameters

**Figure 3:**
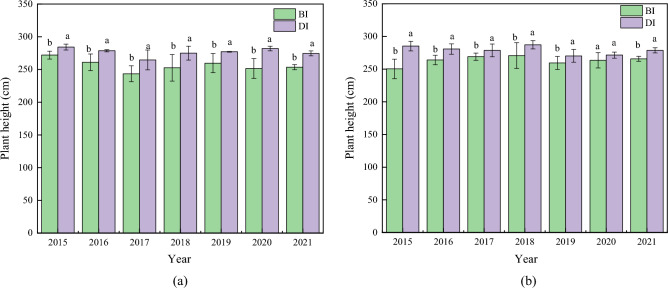
Plant height between drip irrigation and conventional border irrigation at flowering and maturity stages of maize. (**a**) Plant height at flowering stage of maize. (**b**) Plant height at maturity of maize.

**Figure 4:**
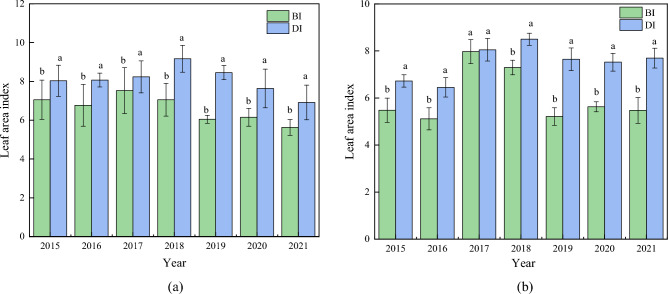
Leaf area index between drip irrigation and conventional border irrigation at flowering and mature stages of maize. (**a**) Leaf area index at flowering stage of maize. (**b**) Leaf area index at maturity of maize.

**Figure 5:**
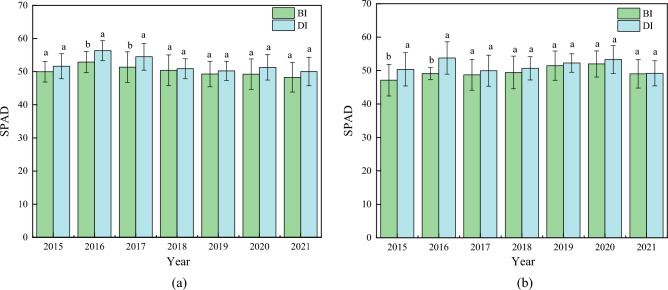
SPAD between drip irrigation and conventional border irrigation at flowering and maturity stages of maize. (**a**) SPAD at flowering stage of maize. (**b**) SPAD at maturity of maize.

Drip irrigation affects maize growth index in different degrees. The plant height, leaf area index and SPAD of average DI maize were higher than those of BI. In comparison to BI, plant height at flowering stage of drip irrigation increased by 7.92%, and that at maturity period increased by 5.95% (Fig. 3). Leaf area index showed that DI was 22.24% higher than BI at flowering stage. At maturity stage, DI was 24.70% higher than BI (Fig. 4). The SPAD of drip irrigation increased by 3.82% and 3.65% compared with BI at flowering stage and maturity stage (Fig. 5). In general, different irrigation methods had great influence on plant height and leaf area index of maize, and there was no significant difference in SPAD between different treatments. Different irrigation methods have specific relationships with maize plant height, leaf area index and SPAD.

### Dry matter accumulation

**Figure 6:**
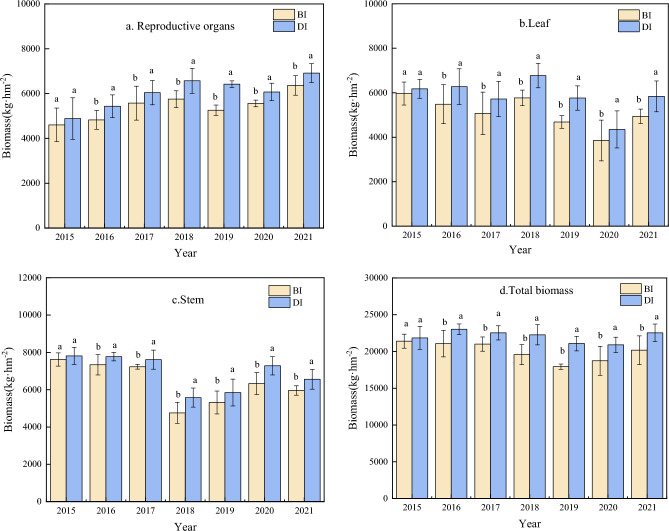
Dry matter accumulation at flowering stage of maize between drip irrigation and conventional border irrigation.

**Figure 7:**
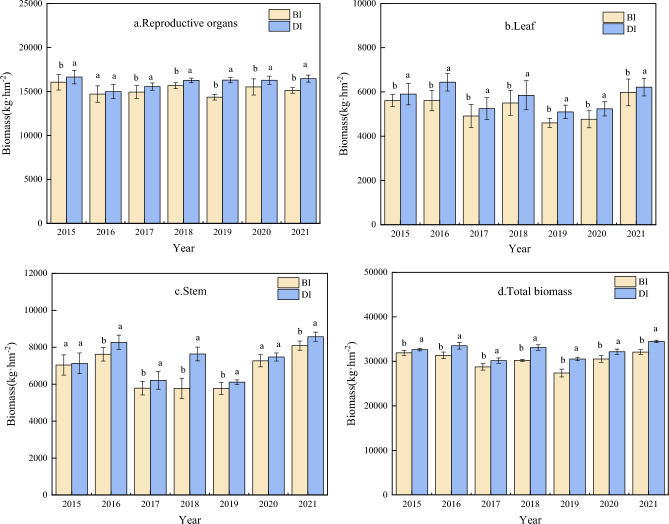
Dry matter accumulation at maturity stage of maize between drip irrigation and conventional border irrigation.

Irrigation methods significantly affected the biomass accumulation of maize at flowering and maturity stages (p < 0.05). In comparison to BI, the reproductive organs biomass of drip irrigation increased by 11.61%, the stems biomass of drip irrigation increased by 8.79%, the leaves biomass of drip irrigation increased by 14.31%, and that the total biomass increased by 10.20% at flowering stage, respectively (Fig. 6). Dry matter accumulation of maize at maturity stage is shown in Fig. 7. The reproductive organs biomass under DI was higher than BI by 5.78%, the leaves biomass increased by 8.17% the stem biomass increased by 8.55% and total biomass increased by 6.75%, respectively. The reproductive organs, leaves, stems and total biomass of maize at maturity stage were higher than those of BI.

### Maize yield and its components

The results in Table 4 demonstrate that, with the exceptions of row number per ear, drip irrigation significantly influenced the maize yield and their components. It can be seen that the yield of maize DI was significantly higher than that under BI. The average yield of maize DI was 1644.51 kg hm^−2^ higher than that under BI, and the increasing rate was 14.39%. In terms of yield structure, ear diameter, kernel number per row, row number per ear and 1000-grain weight showed that drip irrigation was greater than conventional border irrigation, and yield components increased significantly by 2.69%, 8.97%, 2.84% and 7.87%.

**Table 4:** Yield and its component of maize between drip irrigation and conventional border irrigation.

Year	Treatment	Ear diameter (mm)	Kernel number per row	Row number per ear	1000-kernel weight (g)	Yield (kg hm^−2^)
2015	BI	46.21 ± 2.35b	29.37 ± 3.09b	12.60 ± 0.75a	365.55 ± 34.03b	14,916.40 ± 2026.18b
	DI	48.05 ± 3.19a	33.35 ± 1.95a	12.80 ± 0.88a	393.92 ± 21.86a	16,515.66 ± 1617.32a
2016	BI	43.07 ± 2.47b	30.85 ± 1.09b	13.65 ± 1.09a	337.94 ± 17.84b	14,558.23 ± 1663.62b
	DI	44.00 ± 1.61a	33.41 ± 1.14a	14.35 ± 1.41a	365.43 ± 26.76a	16,843.50 ± 1634.13a
2017	BI	46.62 ± 2.58a	30.05 ± 1.44b	14.65 ± 1.55a	305.75 ± 15.16a	14,877.88 ± 1809.44b
	DI	47.01 ± 4.11a	34.20 ± 1.73a	14.94 ± 1.68a	311.80 ± 27.56a	16,739.75 ± 1275.74a
2018	BI	45.55 ± 2.23b	31.66 ± 3.91b	13.84 ± 0.33a	347.23 ± 18.91b	14,622.08 ± 1095.94b
	DI	46.25 ± 2.73a	36.45 ± 2.25a	14.30 ± 0.50a	380.01 ± 25.04a	18,303.94 ± 1218.56a
2019	BI	46.84 ± 1.52b	33.37 ± 3.27a	15.16 ± 0.83a	326.50 ± 72.73b	16,408.04 ± 1614.84b
	DI	47.42 ± 1.94a	34.75 ± 2.52a	15.30 ± 0.69a	353.43 ± 31.95a	16,980.42 ± 1357.87a
2020	BI	47.21 ± 1.77b	31.32 ± 0.21a	13.76 ± 0.50a	356.14 ± 33.57b	17,481.98 ± 1652.32b
	DI	49.55 ± 1.54a	32.35 ± 0.56a	14.64 ± 0.53a	379.81 ± 59.09a	18,800.32 ± 1653.87a
2021	BI	43.63 ± 1.36b	30.55 ± 1.95b	13.95 ± 0.73a	329.50 ± 21.07b	15,072.31 ± 1179.82b
	DI	45.45 ± 1.83a	32.15 ± 1.93a	14.05 ± 0.53a	370.53 ± 19.05a	19,284.74 ± 1059.19a
Mean	BI	45.59b	31.02b	13.94a	338.37b	15,419.56b
	DI	46.82a	33.81a	14.34a	364.99a	17,638.33a

### Dry matter accumulation and translocation of maize

The results in Table 5 demonstrate that the irrigation methods significantly influenced the biomass transfer and related indicators. The dry matter translocation of DI was 27.44% higher than that of BI. The dry matter transfer efficiency of DI was 59.22–85.04%, and BI on was 49.72–75.63%. The dry matter transfer efficiency of DI was 13.97% higher than that of BI. The dry matter contribution of DI was 7.85% higher than that of BI. In general, drip irrigation was superior to conventional border irrigation in dry matter translocation, dry matter transfer efficiency, and grain contribution, which was more beneficial to improve maize yield.

**Table 5:** Effects of drip irrigation and conventional border irrigation on dry matter accumulation and translocation in maize.

Year	Treatment	Dry matter at maturity(kg hm^−2^)	Dry matter at flowering stage (kg hm^−2^)	Dry matter translocation (kg hm^−2^)	Dry matter transfer efficiency (%)	Grain contribution (%)
2015	BI	31,910.45a	19,403.935b	6755.43b	49.72b	45.29b
	DI	32,637.58a	21,846.825a	8812.73a	62.99a	53.36a
2016	BI	31,366.78b	20,076.92b	6843.84b	53.38b	47.01b
	DI	33,221.27a	23,019.3855a	8317.26a	59.22a	49.38a
2017	BI	28,756.98b	19,013.11b	8312.11b	67.54b	55.87b
	DI	30,168.45a	21,534.2a	10,074.93a	75.58a	57.98a
2018	BI	30,227.94b	17,577.34b	6300.88b	59.84b	43.09b
	DI	33,100.56a	22,272.85a	8785.99a	71.13a	48.00a
2019	BI	27,389.34b	17,933.60b	7562.36b	75.63b	46.09b
	DI	30,545.60a	21,079.87a	9872.53a	85.04a	50.12a
2020	BI	30,537.68b	18,734.50b	6707.04b	65.90a	38.37b
	DI	32,175.95a	20,892.99a	8179.79a	70.32a	41.31a
2021	BI	32,099.77b	20,078.46b	6008.71b	55.13b	39.87a
	DI	34,477.40a	22,544.66a	7752.56a	62.54a	40.20a
Mean	BI	30,326.99b	18,973.98b	6927.19b	61.02b	45.08b
	DI	32,332.40a	21,884.40a	8827.97a	69.55a	48.62a

Different letters in the same column indicate significant difference among treatments (*p* < 0.05). The same below.

### Water use efficiency (WUE)

Water use efficiency (WUE) is the standard for comparing the economy of agricultural water use units under different irrigation methods (Table 6). Compared with BI, the IWUE of DI increased 0.99, 1.10, 1.15, 1.36, 1.37, 1.24 and 1.48 kg m^−3^, respectively. The WUE of DI in 7 years increased by 0.82, 0.88, 0.89, 1.18, 1.12, 1.14 and 1.36, which was 53.77% higher that of BI. The average IWUE of DI was 57.90% higher than that of BI. Drip irrigation improved the WUE and IWUE of maize, and played a significant role in saving water.

**Table 6:** Water use efficiency between drip irrigation and conventional border irrigation for maize.

Year	Treatment	Irrigation amount in maize growth period (m^3^ hm^−2^)	Yield 1(kg hm^−2^)	IWUE (kg m^−3^)	WUE (kg m^−3^)
2015	BI	7200	14,916.40b	2.07b	1.89b
	DI	5400	16,515.66a	3.06a	2.71a
2016	BI	7200	14,558.23b	2.02b	1.80b
	DI	5400	16,843.50a	3.12a	2.68a
2017	BI	7200	14,877.88b	2.07b	1.80b
	DI	5400	17,375.97a	3.22a	2.69a
2018	BI	7200	14,622.08b	2.03b	1.87b
	DI	5400	18,303.94a	3.39a	3.05a
2019	BI	7200	16,408.04b	2.28b	2.04b
	DI	5400	19,698.42a	3.65a	3.16a
2020	BI	7200	17,481.98b	2.43b	2.33b
	DI	5400	19,800.32a	3.67a	3.47a
2021	BI	7200	15,072.31b	2.09b	2.00b
	DI	5400	19,284.74a	3.57a	3.36a
Mean	BI	7200	15,419.56b	2.14a	1.96b
	DI	5400	18,260.36a	3.38b	3.01a

### Economic benefits

After deducting the total cost, the net income of DI is significantly higher than BI (Table 7). 7-year gross cost based on the same criteria. Gross cost of BI is higher than that of DI by 3.89%. In comparison to BI, gross return, net returns and benefit/cost ratio (B:C) of DI increased by 18.50%, 60.90% and 22.88%. The net economic returns were calculated by subtracting the inputs from the outputs. The inputs include land-leasing costs, seeds, chemical fertilizers, pesticides, machinery, and labor. Drip irrigation costs less than conventional border irrigation 91.41 USD$ hm^−1^, net income more than 756.58 USD$ hm^−1^.

**Table 7:** Economic analysis between drip irrigation and conventional border irrigation for maize (Amount in USD$ hm^−1^).

Year	Treatment	Gross cost (C)	Gross return (B)	Net return	Benefit/cost ratio (B:C)
2015	BI	2352.44a	3202.28b	849.83b	1.36b
	DI	2261.03b	3545.61a	1284.57a	1.57a
2016	BI	2352.44a	3226.20b	873.76b	1.37b
	DI	2261.03b	3732.63a	1471.60a	1.65a
2017	BI	2352.44a	3090.97b	738.53b	1.31b
	DI	2261.03b	3609.97a	1348.93a	1.60a
2018	BI	2352.44a	3240.35b	887.91b	1.38b
	DI	2261.03b	4056.27a	1795.24a	1.79a
2019	BI	2352.44a	4045.20b	1692.75b	1.72b
	DI	2261.03b	4856.40a	2595.37a	2.15a
2020	BI	2352.44a	4600.52b	2248.08b	1.96b
	DI	2261.03b	5210.61a	2949.58a	2.30a
2021	BI	2352.44a	3757.64b	1405.20b	1.60b
	DI	2261.03b	4807.83a	2546.80a	2.13a
Mean	BI	2352.44a	3594.74b	1242.29b	1.53b
	DI	2261.03b	4259.90a	1998.87a	1.88a

## Discussion

### Effects of irrigation methods on yield and growth indexes of maize

Compared with conventional border irrigation, drip irrigation has the characteristics of short irrigation cycle^26^ and moderate soil moisture obvious dry–wet interface^27^, which is conducive to the growth of maize. Drip irrigation promotes maize growth and increases yield compared with conventional border irrigation. This study showed that compared with conventional border irrigation, drip irrigation increased the average total biomass by 10.20% and 6.75% at flowering and maturity stages, and increased the yield by 10.67%. Sandhu et al.^28^ found that maize and wheat under drip irrigation system showed significant grain yield increase of 13.7% and 23.1% compared to furrow irrigation, respectively. Xu et al.^29^ found the lowest grain yield from the rainfed maize, whereas the drip irrigation method increased grain yield by 14% at 40% water saving than conventional border irrigation. Zhang et al.^30^ reported that drip irrigation under film significantly increased the biomass of maize compared with traditional irrigation, and the biomass at mature stage increased by 6.90%. Li et al.^31^ study found that drip irrigation increased dry matter in growth period and biomass in mature period increased by 4.9–11.1%. The results of previous studies are similar to those of this study. Drip irrigation technologies adopt the advantages of drip irrigation and film mulch, thus creating appropriate crop growth conditions in arable soil layers^32^. Through high-frequency irrigation, drip irrigation slowly applies a small amount of water to the root of the crop, so that the crop is always under better water, avoiding the periodic excessive water and water deficit caused by traditional irrigation^33^. Therefore, drip irrigation is more conducive to maize growth. The results of maize growth and yield in this study are similar to those of previous studies. In sum up, drip irrigation was more beneficial to maize growth and yield increase, the main reasons are: (1) different irrigation methods. Drip irrigation under film changed the water supply site and irrigation frequency, thus affecting the infiltration mode and distribution characteristics of irrigation water, increasing the effective water content in maize root zone, which was beneficial to maize growth and thus increased maize yield. (2) Different irrigation quotas. Many previous studies have shown that the best irrigation quota for maize in northern Xinjiang is about 540 mm, too much or too little is not conducive to the improvement of yield. (3) Different irrigation and fertilization time intervals. The increase of irrigation frequency increased the surface area and weight of the lower root, moved the root outward, increased the volume of ellipsoid, and promoted the growth of maize.

### Effects of irrigation methods on WUE of maize

Drip irrigation drips water and fertilizer directly and slowly into the soil of crop roots through high-frequency irrigation^34^, forming ellipsoidal or spherical wet bodies in the root zone, which is beneficial for crops to absorb water from the soil and can effectively reduce deep leakage^35^. Because the water of drip irrigation is mainly distributed in the root zone of maize, it is beneficial to the absorption and utilization of crops, thus improving the water use efficiency^36^. Therefore, drip irrigation has better WUE and IWUE than conventional border irrigation for maize. The WUE of crops is an important index to measure the water absorption and utilization efficiency of crops. This study shows that the average WUE of drip irrigation is 3.01%, and that of conventional border irrigation is 1.96%, and the average IWUE of drip irrigation is 3.38%, and that of conventional border irrigation is 2.14%, and the water production efficiency is increased by 57.90%. The water production efficiency is increased by 53.77%. Xiong et al.^37^ studies have shown that intermittent irrigation increases water use efficiency by at least 18.2% compared with conventional irrigation. Rasool et al.^38^ found that compared with the furrow irrigation treatment, water savings of 33.4–60.0% were found under drip irrigation treatments. Ghamarnia et al.^39^ showed that compared with the local conventional furrow irrigation, the seasonal irrigation water of drip irrigation maize was saved by 36–81% by using different drip irrigation belts and surface treatments, combined with soil and water monitoring. Fonteyne et al.^40^ showed that under conventional tillage conditions, drip irrigation water saving 36% than furrow irrigation on average, drip irrigation and conservation agriculture combined irrigation water saving 40% than furrow irrigation on average. Those were consistent with our results. Drip irrigation not only improves the irrigation water use efficiency but also achieves the effect of water-saving irrigation. Significant economic, social and ecological benefits have been obtained by the large-scale popularization and application of drip irrigation technology in northwest China.

## Conclusions

Under the condition of basic irrigation amount in northwest China, drip irrigation promoted maize growth, dry matter accumulation and WUE compared with conventional border irrigation, thereby increasing yield. Compared with conventional border irrigation, drip irrigation is a saving water, high yield and high efficiency irrigation method for maize planting in northwest China. The yield of maize under drip irrigation was 14.39% higher than that of conventional border irrigation, the water use efficiency was increased by 43.48–68.09%, the irrigation water use efficiency was increased by 47.63–70.60%, and economic efficiency increase of 756.58USD$ hm^−1^. Drip irrigation become a more effective measure for sustainable agricultural development in the future, which has cut down on irrigation water about 180 mm.

## Data Availability

The datasets used and/or analysed during the current study available from the corresponding author on reasonable request.

## References

[1] Nassab ADM, Amon T, Kaul HP (2011). Competition and yield in intercrops of maize and sunflower for biogas. Ind. Crops Prod. Sci..

[2] Jin W, Liu SS, Zhang K, Kong W (2018). Influence of agricultural production efficiency on agricultural water consumption. J. Nat. Resour..

[3] Niu Z, Zhang Y, Li T, Balezentis T, Shen Z (2021). Total factor productivity growth in China’s corn farming: An application of generalized productivity indicator. Bus. Econ. Manage..

[4] Yan S, Wu Y, Fan J, Zhang F, Qiang S, Zheng J, Xiang Y, Guo J, Zou H (2019). Effects of water and fertilizer management on grain filling characteristics, grain weight and productivity of drip-fertigated winter wheat. Agric. Water Manage..

[5] Wang H, Wu L, Cheng M, Fan J, Zhang F, Zou Y, Chau HW, Gao Z, Wang X (2018). Coupling effects of water and fertilizer on grain yield, water and fertilizer use efficiency of drip-fertigated cotton in northern Xinjiang. China. Field Crops Res..

[6] Joshua E, Delphine D, Christoph M, Katja F, Markus K, Dieter G, Michael G, Martina FR, Yoshihide W, Neil B (2014). Constraints and potentials of future irrigation water availability on agricultural production under climate change. Proc. Natl. Acad. Sci. USA.

[7] Mitchell AR, Van Genuchten MT (1993). Flood irrigation of a cracked soil. Soil Sci. Soc. Am. J..

[8] Wang Y, Li S, Cui Y, Qin S, Guo H, Yang D, Wang C (2021). Effect of drip irrigation on soil water balance and water use efficiency of maize in Northwest China. Water.

[9] Suryavanshi P, Buttar GS (2020). Influence of different irrigation methods and schedules on water productivity of wheat. J. Soil Water Conserv..

[10] Zhang TB, Zou YF, Kisekka I, Biswas A, Cai HJ (2021). Comparison of different irrigation methods to synergistically improve maize’s yield, water productivity and economic benefits in an arid irrigation area. Agric. Water Manage..

[11] Liu HJ, Yuan BZ, Hu XD, Yin CY (2021). Drip irrigation enhances water use efficiency without losses in cucumber yield and economic benefits in greenhouses in North China. Irrig. Sci..

[12] Raina JN, Thakur BC, Verma ML (1999). Effect of drip irrigation and polyethylene mulch on yield, quality and water use efficiency of tomato. Indian J. Agric. Sci..

[13] Michel JC, Kerloch E (2017). Evolution of hydraulic properties and wettability of organic growing media during cultivation according to irrigation strategies. Sci. Hortic..

[14] Zhu ZR, Zhu DL, Ge MS, Liu CX (2022). Effects of the growing-maize canopy and irrigation characteristics on the ability to funnel sprinkler water. J. Arid Land..

[15] Qin SJ, Li S, Kang SZ, Du TS, Tong L, Ding RS (2016). Can the drip irrigation under film mulch reduce crop evapotranspiration and save water under the sufficient irrigation condition?. Agric. Water Manage..

[16] O’Neill CJ, Humphreys E, Louis J, Katupitiya A (2008). Maize productivity in southern New South Wales under furrow and pressurised irrigation. Aust. J. Exp. Agric..

[17] Mehriya ML, Geat N, Sarita X, Singh H, Mattar MA, Elansary HO (2020). Response of drip irrigation and fertigation on cumin yield, quality, and water-use efficiency grown under arid climatic conditions. Agronomy Basel.

[18] Gong LS, Qu SJ, Huang GM, Guo YL, Zhang MC, Li ZH, Zhou YY, Duan LS (2021). Improving maize grain yield by formulating plant growth regulator strategies in North China. J. Integrative Agric..

[19] Zheng J, Fan JL, Zhang FC, Yan SC, Xiang YZ (2018). Rainfall partitioning into throughfall, stemflow and interception loss by maize canopy on the semi-arid Loess Plateau of China. Agric. Water Manage..

[20] Smoczynska A, Szweykowska-Kulinska Z (2016). MicroRNA-mediated regulation of flower development in grasses. Acta Biochim. Pol..

[21] EL-Hendawy SE, EL-Lattief EAA, Ahmed MS, Schmidhalter U (2008). Irrigation rate and plant density effects on yield and water use efficiency of drip-irrigated corn. Agric. Water Manage..

[22] Cox MC, Qualset CO, Rains DW (1985). Genetic variation for nitrogen assimilation and translocation in wheat. II. Nitrogen assimilation in relation to grain yield and protein. Crop Sci..

[23] Bandyopadhyay, K. K. *et al.* (2009). Effect of irrigation and nitrogen application methods on input use efficiency of wheat under limited water supply in a vertisol of central india. *Irrigation Science* **28**(4), 285–299.

[24] Guizani M, Dabbou S, Maatallah S, Montevecchi G, Hajlaoui H, Rezig M, Helal AN, Kilani-Jaziri S (2019). Physiological responses and fruit quality of four peach cultivars under sustained and cyclic deficit irrigation in center-west of Tunisia. Agric. Water Manage..

[25] Geng YH, Cao GJ, Wang LC, Wang M, Huang JX (2020). Can drip irrigation under mulch be replaced with shallow-buried drip irrigation in spring maize production systems in semiarid areas of northern China?. J. Sci. Food Agric..

[26] Ma HH, Yang T, Niu XX, Hou ZN, Ma XW (2021). Sound water and nitrogen management decreases nitrogen losses from a drip-fertigated cotton field in northwestern china. Sustainability.

[27] Entz MH, Gross KG, Fowler DB (1992). Root growth and soil-water extraction by winter and spring wheat. Can. J. Plant Sci..

[28] Sandhu OS, Gupta RK, Thind HS, Jat ML, Sidhu HS, Yadvinder-Singh X (2019). Drip irrigation and nitrogen management for improving crop yields, nitrogen use efficiency and water productivity of maize-wheat system on permanent beds in north-west India. Agric. Water Manage..

[29] Xu S, Wei YC, Laghari AH, Yang XM, Wang TC (2021). Modelling effect of different irrigation methods on spring maize yield, water and nitrogen use efficiencies in the North China Plain. Math. Biosci. Eng..

[30] Zhang YQ, Wang JD, Gong SH, Xu D, Sui J, Wu ZD (2018). Analysis of water saving and yield increasing mechanism in maize field with drip irrigation under film mulching based on transpiration estimated by sap flow meter. Trans. Chin. Soc. Agric. Eng..

[31] Li ZJ, Yang JJ, Fan FF, Hou YP, Xie JG, Liang YC (2011). Effect of plastic film mulching on dry mass accumulation and phosphorus uptake of corn receiving different fertilizers. Plant Nutr. Fertil. Sci..

[32] Zhang GQ, Liu CW, Xiao CH, Xie RZ, Ming B, Hou P, Liu GZ, Xu WJ, Shen DP, Wang KR, Li SK (2017). Optimizing water use efficiency and economic return of super high yield spring maize under drip irrigation and plastic mulching in arid areas of China. Field Crops Res..

[33] Gui YP, Wang QM, Zhao Y, Ma MY, Li HH, Zhai JQ, Li EC (2022). On the increased precipitation recycling by large-scale irrigation over the Haihe Plain. J. Meteorol. Res..

[34] Saeed IAM, EI-Nadi AH (1998). Forage sorghum yield and water use efficiency under variable irrigation. Irrig. Sci..

[35] Qi DL, Hu TT, Song X (2020). Effects of nitrogen application rates and irrigation regimes on grain yield and water use efficiency of maize under alternate partial root-zone irrigation. J. Integr. Agric..

[36] Ding YT, Cheng Y, Zhang TB, Ji XX, Qiao RN, Feng H (2021). Modeling of dynamics of deep soil water and root uptake of maize with mulched drip irrigations using HYDRUS-2D. Agric. Res. Arid Areas.

[37] Xiong RY, Xie JX, Tan XM, Yang TT, Pan XH, Zeng YJ, Shi QH, Zhang J, Cai S, Zeng YH (2021). Effects of irrigation management on grain yield and quality of high-quality eating late-season indica rice in South China. Sci. Agric. Sin..

[38] Rasool G, Guo XP, Wang ZC, Ullah I, Chen S (2020). Effect of two types of irrigation on growth, yield and water productivity of maize under different irrigation treatments in an arid environment. Irrig. Drain..

[39] Ghamarnia H, Parandyn MA, Arji I, Rezvani V (2013). An evaluation and comparison of drip and conventional furrow irrigation methods on maize. Arch. Agron. Soil Sci..

[40] Fonteyne S, Garcia AF, Verhulst N (2022). Reduced water use in barley and maize production through conservation agriculture and drip irrigation. Front. Sustain. Food Syst..

